# The Effect of the Rice Endosperm Protein Hydrolysate on the Subjective Negative Mood Status in Healthy Humans: A Randomized, Double-Blind, and Placebo-Controlled Clinical Trial

**DOI:** 10.3390/nu15153491

**Published:** 2023-08-07

**Authors:** Ryoko Nakayama, Daisuke Nishi, Masaru Sato, Akira Ito, Kimiko Uchiyama, Yuki Higuchi, Hajime Takahashi, Kousaku Ohinata

**Affiliations:** 1 Rice Research Institute, Kameda Seika Co., Ltd., Niigata 950-0198, Niigata, Japan; 2 Department of Mental Health, Graduate School of Medicine, The University of Tokyo, Tokyo 113-0033, Japan; d-nishi@m.u-tokyo.ac.jp; 3 Department of Applied Genomics, Kazusa DNA Research Institute, 2-6-7 Kazusakamatari, Kisarazu 292-0818, Chiba, Japan; msato@kazusa.or.jp; 4 Division of Food Science and Biotechnology, Graduate School of Agriculture, Kyoto University, Uji 611-0011, Kyoto, Japan; ohinata.kousaku.3n@kyoto-u.ac.jp

**Keywords:** functional food, mood state, rice endosperm protein, Euthymia Scale, POMS 2

## Abstract

The rice endosperm protein (REP) hydrolysate containing the following rice endosperm protein derived oligopeptides QQFLPEGQSQSQK, LPEGQSQSQK, and pEQFLPEGQSQSQK (a *N*-terminal pyroglutamate residue-modified peptide) reportedly showed an antidepressant-like effect in an animal model. We investigated the effect of the REP hydrolysate on healthy humans who self-reported mental fatigue with subjectively low vigor. Seventy-six participants (age: 20–64 years) were randomly allocated to two groups. The influence of the REP hydrolysate on the mood state was evaluated in two studies: single intake (Study 1) and repeated intake over 4 weeks (Study 2). A salivary stress marker, Chromogranin A (CgA), was measured in Study 1. The single intake of the REP hydrolysate significantly improved the Profile of Mood Status 2nd edition for adults (POMS 2) subscale of Tension–Anxiety. Additionally, the salivary CgA concentrations were remarkably reduced after the single intake of the REP hydrolysate. Though a single intake of the REP hydrolysate did not significantly influence the other subscales and the TMD of the POMS 2 and the Euthymia Scale, both the subjective and objective results supported the possible effect of the REP hydrolysate on reducing anxiety and nervousness. No significant positive effects on the subjective mood state (Euthymia Scale and POMS 2) and sleep quality (Insomnia Severity Index) were observed in the trial setting employed for Study 2. In conclusion, a single intake of REP hydrolysate might help relax the subjective feelings of tension and anxiety. The effectiveness of repeated REP hydrolysate intake needs to be tested in a different clinical setting.

## 1. Introduction

Rice (*Oryza sativa*) is consumed by approximately half of the world’s population [1]. It is well known that rice is one of the nutritionally important foods for humans, especially as a source of carbon hydrates. Not as well known, rice is also a great source of protein. Rice consists of 88% endosperm, 10% bran, and 2% germ [2]. The majority of consumers prefer well-peeled rice containing the remaining endosperm and little or no bran [3,4]. The removal of the outer layers of rice indeed reduces some nutrition content compared to non-peeled rice; however, water soluble nutrients can spread into the endosperm during the cooking process. Hence, milled rice can contain more water-soluble nutrition [5]. Considering that rice is often consumed in the form of peeled rice, the frequently consumed protein from rice is the rice endosperm protein (REP). Indeed, a survey found that 10% of the daily protein intake comes from rice among the Japanese population aged over 20, which is the third most dominant protein source after meat and fish [6].

REP accounting for approximately 5% of rice endosperm is not only nutritionally important but also shows various physiological functions such as anti-hyperglycemia, anti-hyperuricemia, and anti-obesity when orally administrated [7,8,9]. Recently, there have been various peptides derived from food protein resources reported to exhibit physiological functions [10,11]. With regard to peptides, soy-protein-derived peptides have been reported to show anti-hyperglycemia, an opioid-like effect, and an orexigenic effect with smaller doses than proteins [12,13]. REP, on the other hand, is still a novel source of peptides. Based on the research on the functionality of REP, it seems promising that the REP provides physiologically active peptides. Even though various functionalities of REP have been reported, REP has not been provided much as a functional food because of its high dose (e.g., 10 g/day). Thus, turning REP into a functional ingredient that can be physiologically active with a small dose, namely functional peptides, would be a truly meaningful approach.

Mental illness threatens many populations today. A great number of people are reported to suffer from psychological distress [14,15]. Untreated psychological distress can progress to severe health problems, such as depression and anxiety, which can ultimately ruin an individual’s quality of life [16,17,18]. Antidepressants can be used to treat mental distress; however, cases in which such interventions show low (or no) effects on patients with mild or moderate symptoms are reported to be problematic [19]. Furthermore, certain groups of people, such as pregnant women, tend to be hesitant to use psychoactive drugs [20]. Thus, other types of intervention are needed. Nutritional intervention, for example, may be a safe and accessible remedy [21,22,23,24,25].

Rice endosperm protein hydrolysate (REP hydrolysate) containing the following rice oligo peptides; QQFLPEGQSQSQK, LPEGQSQSQK, and pEQFLPEGQSQSQK (a *N*-terminal pyroglutamate residue-modified peptide) has recently revealed its strong antidepressant-like effect in an animal model with a single administration [26]. Its effect on humans, however, remains unknown. 

Two clinical studies were designed: Study 1 for the single-dose effect of REP hydrolysate on human mood and Study 2 for the effect of the repeated intake of REP hydrolysate on human mood. The aim of this study was to evaluate the effects of REP hydrolysate consumption on human mood. 

## 2. Materials and Methods

### 2.1. Study Design

This study was performed with a randomized, double-blind, and placebo-controlled design. Study 1 and Study 2 were conducted to evaluate our hypothesis of whether the intake of rice endosperm protein hydrolysate improved the negative mood status of humans when consumed only once (Study 1) and once a day for 4 weeks (Study 2). 

#### 2.1.1. Study 1

Participants visited the study facility on the first test day. After a health check, the Profile of Mood Status 2nd edition for adults (POMS 2) [27] and the Euthymia Scale [28], which are the questionnaires to assess the mood state and the Euthymia state, respectively, were conducted to determine the baseline for Study 1. After 5 min of resting, saliva samples were collected for a biomarker analysis. Participants consumed the test food corresponding to the arm to which they were allocated with a light meal prepared by the clinical research organization. At 3 h after consuming the test food, participants undertook the psychological survey, after which saliva samples were collected. 

The animal study indicated that the positive effect of REP hydrolysate might be observed in a short time. Namely, the administration of REP hydrolysate shortened the immobile time on the tail suspension test by 5 min after a single oral administration. Furthermore, a previous clinical trial (Clinical Trial Registry No. UMIN 000041724) indicated that REP hydrolysate single intake induced a strong effect size of d = 0.8 in inter-group comparison on the Euthymia Scale change. Thus, Study 1 was designed to see whether a single intake of REP hydrolysate had an influence in humans. 

#### 2.1.2. Study 2

Participants completed the POMS 2 and the Euthymia Scale on the third pre-examination day and completed the Insomnia Severity Index (ISI), which is a self-rating questionnaire to assess the subjective severity of insomnia, on the first day of the test food period as baseline evaluations. Participants answered the POMS 2, Euthymia Scale, and ISI once a week during the 4-week test food period. Within 3 days after the final day of the fourth week, participants visited the study facility for a health check and saliva sample collection. Life events were recorded throughout the entire study period.

The study period was set to 4 weeks, because 4 weeks of repeated intake was hypothesized to be long enough to overcome the placebo effect and detect the intervention effect, as a clinical trial that investigated the effect of GABA on the subjective mood state showed the declined placebo effect on the fourth week in the 12 weeks of the trial period and showed significant intervention effect on the fourth week [29].

### 2.2. Procedure

All study procedures were reviewed and approved by the Ethics Committee of Medical Station Clinic (IRB no. 20000022, 8 July 2021) and were conducted in accordance with the criteria set by the declaration of Helsinki. The trial was preregistered at the UMIN Clinical Trial Registry (no. UMIN000045037). Throughout the entire period, this study was conducted according to a previously approved study protocol. This study was operated by EP Mediate Co., Ltd. (Tokyo, Japan) and performed at Iryo Hojin Shadan Kowa Clinic from August 2021 to March 2022.

### 2.3. Participants

Two hundred fifty-one potential participants were informed verbally and documentarily of this study and signed an informed consent form before participation. These candidates went through the following pre-examination sessions. In total, 76 subjects who were healthy and eligible to participate in this study based on the results of the pre-examinations participated in this clinical trial. The eligibility criteria were (1) those who were healthy adults 20–64 years of age and (2) those who scored higher than 50 on the Profile of Mood Status 2nd edition short form for adults (POMS 2) Fatigue–Inertia subscale and less than 50 on the POMS 2 Vigor–Activity subscale at least once in the two pre-examinations. Participants were excluded if they met the following exclusion criteria: (1) those who were under medical treatment due to mental health issues and/or tested positive on a Major Depression Episode Sheet, (2) those who were on night shift or whose lifestyle was day–night reversed, or who went to bed from 6:00 a.m. to 10:00 a.m. and woke up from 6:00 p.m. to 8:00 p.m. at least once per week, (3) those who habitually consumed energy drinks more than once a week, (4) those who saw a doctor, were under medical treatment, or who took medicines/supplements in order to reduce stress and/or improve sleep quality, (5) those who were allergic to or had shown allergic reactions to certain allergens, (6) those with a medical condition that required chronic drug administration or who had a history of malignant tumor that required drug administration except for those who were diagnosed as being in remission, (7) those whose hematology test and vital sign assessment results were categorized as worse than group D, as defined by the Japan Society of NINGEN Dock, (8) those who were currently attending another clinical trial, (9) those who planned to become pregnant or to breastfeed during the study period, and (10) those who the principal investigator decided were unable to undergo the investigations and/or observe the patient restrictions. Seventy-six participants who satisfied the criteria listed above were enrolled in this study and were randomly allocated to the REP hydrolysate arm (*n* = 38) or the placebo arm (*n* = 38). 

### 2.4. Test Foods

The REP hydrolysate was manufactured by Kameda Seika Co., Ltd. (Niigata, Japan). A tablet type food containing REP hydrolysate (25 mg/each) was used as the active food for the REP hydrolysate group. Two tablets were administered per day. Participants were taught to take the daily dosage at once. The concentration of the active peptides or the rice oligo peptides, LPEGQSQSQK, QQFLPEGQSQSQK, and pEQFLPEGQSQSQK (a *N*-terminal pyroglutamate residue-modified peptide), were quantitated at 10 µg/day in total (5.0 µg/day, 2.0 µg/day, and 3.0 µg/day, respectively). The nutrition composition in one active tablet was 0.02 g of protein, 0.005 g of fat, and 0.215 g of carbon hydrate. The total energy was 0.98 kcal. One without REP hydrolysate was used for the placebo group. The nutrition composition of the placebo food was 0.005 g of fat, and 0.235 g of carbon hydrate. The total energy was 0.99 kcal. These tablets were made undistinguishable in their appearance and flavor. 

### 2.5. Measurements

#### 2.5.1. Subjective Mood State Questionnaire

The psychological status was assessed with the following validated questionnaires: the Profile of Mood Status 2nd edition short form for adults (POMS 2) and the Euthymia Scale. POMS 2 is a self-reported questionnaire that assesses mood. It has two categories: the negative mood state and the positive mood state. The negative mood state contains the following five subcategories: Anger–Hostility (AH), Confusion–Bewilderment (CB), Depression–Dejection (DD), Fatigue–Inertia (FI), and Tension–Anxiety (TA). The positive mood state contains Vigor–Activity (VA) and Friendliness (F). The total mood disturbance (TMD) was calculated for the individual participants by subtracting the score of the positive mood from the score of the negative mood. The reliability and validity of the Japanese version of the POMS 2 have been confirmed [30,31].

The Euthymia Scale is a self-rated questionnaire for the self-assessment of euthymia. The reliability and validity of the Japanese version have recently been reported [32]. Euthymia is an idea defined by the existence of positive effects, psychological flexibility, psychological wellbeing, a unifying outlook on life, and resistance to stress (resilience and tolerance to anxiety or frustration), not only by the absence of mood disturbances [33]. The score range of the Euthymia Scale is 0 to 10. Higher points in this scale indicate a better state of euthymia. 

#### 2.5.2. Subjective Sleep Quality Evaluation

The Insomnia Severity Index (ISI) is a questionnaire that measures the subjective severity of insomnia. A higher score indicates more severe insomnia. The reliability and validity of the Japanese version of the ISI were previously confirmed [34].

#### 2.5.3. Measurements of Salivary CgA Concentrations

Saliva was collected with a swab and centrifuge tubes. Collected saliva samples were stored at −80 °C until measurement. The concentration of the salivary Chromogranin A (CgA) was determined with a commercially available human CgA EIA kit (Yanaihara Institute Inc., Shizuoka, Japan). The total protein in the saliva samples was measured by the Bradford method with a commercially available dye-assay kit (Takara Bio Inc., Shiga, Japan). The concentration of CgA was corrected with the amount of salivary total protein.

### 2.6. Outcomes

#### 2.6.1. Study 1

The primary outcome for study 1 was the POMS 2 scores (AH, CB, DD, FI, TA, VA, F, and TMD). The secondary outcome was the Euthymia Scale score and salivary CgA concentration.

#### 2.6.2. Study 2

The primary endpoint was the change in the Euthymia Scale scores from the baseline to the fourth week of intervention. The secondary outcomes were the POMS 2 and ISI scores.

### 2.7. Sample Size Calculation

Based on the results of the previous clinical trial (Clinical Trial Registry no. UMIN 000041724), a total of 66 participants was calculated to be necessary to detect a difference of 1.27, with 1.7 standard deviations in the Euthymia Scale between the REP hydrolysate group and the placebo group with a two-sided significance level of 5% and 85% power. The final sample size was determined to be 76 with a predicted dropout rate of 15%.

### 2.8. Randomization and Blinding

A person in charge of allocation randomly allocated the participants to the REP hydrolysate arm or the placebo arm with the block randomization method. A computationally generated random allocation sequence was used. The block size of the RCT was set to 4. The allocation factors were the POMS 2 score of the FI and VA and the total score of the Euthymia Scale. The person in charge of allocation concealed the allocation table until the day of allocation disclosure and ensured that the participants and the rest of the study members were appropriately blinded to the allocation. 

### 2.9. Statistical Analyses

The results are shown as the mean ± standard error of the mean (SEM). For Study 1, the changes before and after the intake of test food were evaluated. For Study 2, the changes from the baseline to the fourth week were evaluated. The independent *t*-test was used for the statistical analyses. All analyses were performed using IBM SPSS Statistics ver. 27. *p* values of <0.05 (two-tailed) were considered statistically significant. When statistical significance was observed, the effect size was evaluated with the Cohen’s d value. The Cohen’s d values were considered as a trivial effect (d < 0.2), a small effect (0.2 ≤ d < 0.5), a medium effect (0.5 ≤ d < 0.8), and a large effect (d ≥ 0.8) [35]. The Cohen’s d was calculated with the following equations.

E1: Cohen’s d = (M_2_ − M_1_)/SD_pooled,_

E2: SD_pooled_ = √((SD_1_^2^ + SD_2_^2^)/2).

## 3. Results

**Figure 1:**
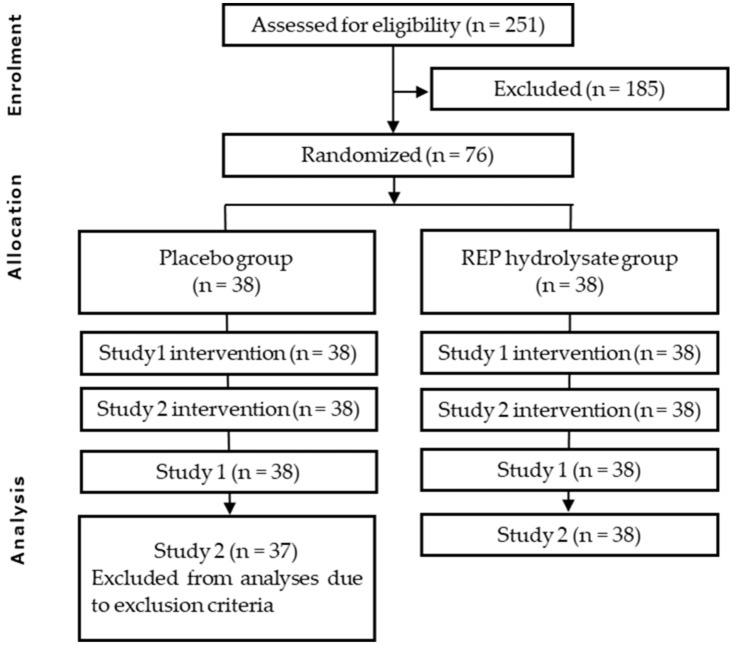
Flow diagram for study participants’ eligibility, enrollment, and followup.

The participants’ enrollment in this study is described in Figure 1. Among the 251 participants who went through the screening stage, 76 participants who satisfied the inclusion criteria and did not meet any of the exclusion criteria were enrolled. The background characteristics of the participants are shown in Table 1. All the variables were measured at the first pre-examination date apart from the baseline score of the POMS 2 and the Euthymia Scale. The values of the POMS 2 and Euthymia Score were measured at the third pre-examination date. There was no statistically significant intergroup difference on the baseline score of the POMS 2 FI, POMS 2 VA, the Euthymia Scale, or the allocation factors. Seventy-six participants were randomly allocated to either the REP hydrolysate arm (Active) or placebo arm (Placebo) and attended Study 1. All 76 participants proceeded to Study 2. Seventy-six participants completed the scheduled intervention. One participant was excluded from the efficiency analysis for Study 2 due to the violation of the participant restrictions or repeated medication treatment during the period of Study 2. Thus, the final per-protocol sets for Study 1 and Study 2 in the efficiency analysis were 76 and 75, respectively. Exclusion was conducted before revealing the allotment list and based on the exclusion criteria set before the trial started. A safety analysis was conducted for all 76 participants. No adverse effects from REP hydrolysate consumption were reported. The overall study schedule is described in Table 2. After three pre-examinations, participants were randomly allocated into placebo or active arms. On the first day, Study 1 was conducted, and then Study 2 followed. Within 3 days after the final date of Study 2, participants visited the hospital for followup. Salivary stress marker analyses were not conducted for Study 2. 

### 3.1. Study 1

#### 3.1.1. Subjective Mood State Questionnaire (POMS 2, Euthymia Scale)

Table 3 and Table 4 show the results of the POMS 2 and Euthymia Scale. Changes from the pre-intake to post-intake were calculated for all the subscales of the POMS 2, TMD, and the total score of Euthymia. The calculated changes were then statistically evaluated between the two groups. Among the calculated changes in the POMS 2, the Tension–Anxiety subscale significantly decreased in the active group in comparison to the placebo group (*p* = 0.037, Cohen’s d = 0.49). The REP hydrolysate consumption was not associated with any significant improvement in the scores of the other POMS subscales, the TMD, or the Euthymia Scale.

#### 3.1.2. Salivary CgA Concentrations (Post Hoc)

As a subjective mood improvement was observed in Study 1, the salivary concentration of CgA was conducted as a post hoc analysis. CgA concentrations were evaluated in 66 of the 76 participants (placebo, *n* = 30; active, *n* = 36). Ten participants were excluded from the salivary CgA concentration analysis because the amount of CgA in the saliva samples was insufficient (*n* = 3) or because the amount of saliva was insufficient for the salivary CgA concentration assay (*n* = 7). Table 5 shows the results of the salivary CgA concentration assay. The change from pre-intake to post-intake was calculated, and the change in the active group was greater than that in the placebo group. The inter-group comparison of the change showed a statistical significance with a moderate effect size (*p* = 0.034, Cohen’s d value = 0.54). No statistically significant difference was observed in the baseline CgA concentrations of the two groups. No other markers were analyzed.

### 3.2. Study 2

#### 3.2.1. Subjective Mood State Questionnaire (POMS 2, Euthymia Scale)

Table 6 and Table 7 show the results of the POMS 2 and the Euthymia Scale during the 4-week trial. The changes from the baseline to after-4 week point were evaluated. There was no positive significant treatment effect on the REP hydrolysate group in comparison to the placebo group on any of the conducted questionnaires.

#### 3.2.2. Subjective Insomnia Severity

Table 8 shows the results of the ISI. Changes from the baseline to after-4 week point were evaluated. There was no significant positive effect on the REP hydrolysate consumption.

## 4. Discussion

This study was conducted to confirm the effect of the single intake and repeated intake of REP hydrolysate on the subjective mood. The single intake of REP hydrolysate significantly improved the POMS 2 subscale of Tension–Anxiety (*p* = 0.037, Cohen’s d = 0.49). Additionally, the salivary CgA concentrations were remarkably reduced on the single intake of REP hydrolysate (*p* = 0.034, Cohen’s d = 0.54). Though the single intake of REP hydrolysate did not significantly influence the other subscales, the TMD of POMS 2, or the Euthymia Scale, both the subjective and objective results supported the effect of REP hydrolysate on reducing anxiety and nervousness. The repeated intake of REP hydrolysate over 4 weeks did not show any significant positive effect on the subjective mood state (Euthymia Scale and POMS 2) and sleep quality (ISI) in the trial setting employed for this study. From this study, though the effectiveness of repeated REP hydrolysate intake needs to be tested in a different clinical setting, it was suggested that a single intake of REP hydrolysate might help relax the subjective feelings of tension and anxiety through the autonomic nerve system. 

The single intake of REP hydrolysate showed a significant improvement in the POMS 2 Tension–Anxiety subscale (*p* = 0.037, Cohen’s d = 0.49). Based on the classic classification of Cohen’s d, the d value detected in Study 1 was a small effect. However, considering that food ingredients tend to have a small effect size and even many of modern antidepressants can only show effect size of d = 0.3 [36], the intervention effect of the Cohen’s d = 0.49 found in POMS 2 TA in Study 1 may be notable. The single intake of REP hydrolysate did not improve the other POMS 2 subscales and the Euthymia Scale. The animal study showed that the vagotomy treatment partially canceled the effect of the single intake of REP hydrolysate on the tail suspension test [26]. These findings indicated that orally consumed REP hydrolysate interacted with the gastrointestinal surface, and its signal was transmitted to the brain through the vagus nerve system, which is independent of intestinal absorption. For example, matured hop bitter acid has been reported to promote the secretion of cholecystokinin, a gastrointestinal hormone, from the enteroendocrine cells of the duodenum and jejunum. The CCK signal is transmitted through the vagus nerve and norepinephrine is induced in the brain [37,38]. A similar mechanism via gut–brain communication may relax anxiety and nervousness through the consumption of REP hydrolysate. The vagus nerve reaction is known as a rapid reaction [39,40]. Thus, this clinical result showing the short-term effect of REP hydrolysate does not contradict the previous findings. 

As a subjective mood improvement was observed in Study 1, the salivary concentration of the CgA was analyzed. The salivary CgA was selected because it reportedly reduces its concentration by anxiolytic ingredients, reflects acute psychological stress, has a relatively small daytime disturbance, and is rarely influenced by physical movement [41,42,43,44]. CgA is an acidic glycoprotein that is released together with catecholamines from the adrenal medulla, which reflects the activation of the sympathetic nerve system [45]. Little is known about its correlation with depression and anxiety; this compound has been reported to be a promising stress marker [46,47]. The single intake of REP hydrolysate inhibited an increase in the salivary CgA concentration with a significant medium size effect (*p* = 0.034, Cohen’s d = 0.54). From this result, the single intake of REP hydrolysate seemed to calm the sympathetic nerve system and might cause shifts to a parasympathetic nerve dominant state, which possibly leads to the easing of the degree of subjective feelings of anxiety and nervousness. Hence, the objective measurement of the salivary CgA concentration also supported the effect of REP hydrolysate in the reduction in subjective feelings of anxiety and nervousness. 

The reason why no significant positive intervention effect of REP hydrolysate after the 4-week intake was observed might be because the duration of intervention was insufficient. As mentioned above, the animal study showed that the vagotomy treatment partially inhibited the effect of REP hydrolysate on the tail suspension test. This implies that the REP hydrolysate achieved an antidepressant-like effect through the humoral pathway, in addition to the neural pathway. For example, it is known that blood pressure is dually regulated by neural regulation and humoral regulation. Furthermore, it is controlled with neural regulation for the short term, whereas it is controlled with humoral regulation for the long term [48]. Some food ingredients with mood-enhancing effects, which were implied to have humoral mechanisms, were tested for their effectiveness over a longer period. The effectiveness of saffron extract, one of the food ingredients that was reported to show an antidepression and an anxiolytic effect, was clinically confirmed with 8–12 weeks of intervention [24,49,50,51]. Thus, for the humoral mechanism, it may require a longer intervention period than 4 weeks. 

Previous research reported that not only stressors in the past but also anticipating upcoming stressors can negatively affect the current status [52,53]. During Study 2, the participants were asked to self-evaluate their mood status over the past week, including the evaluation time point at which they were asked to rate their current mood in Study 1. Consequently, the dataset obtained on the subjective mood status from Study 2 can be translated to how retrospective stressors affected the current mood. Thus, a trial setting that involves stressor anticipation might be a suitable way to evaluate the favorable effects of REP hydrolysate on humans. Therefore, the current design for Study 2 might have not been suitable to see the effect of REP hydrolysate on human mood. 

Despite this study’s limitations, including the modest sample size, the POMS 2 validation confirmed only with younger population, the lack of information that could affect subjective mood states (e.g., past history of mental disorder and marriage status), the number of participants that were excluded from the salivary analysis, and no experiment-wide false detection rate evaluated, this study obtained positive results under the best conditions that could be achieved.

## 5. Conclusions

In the present study, the 4-week intake of REP hydrolysate did not show a positive effect, possibly due to the insufficient length of intervention. The single intake of REP hydrolysate did not improve the Euthymia Scale or all of the subscales of POMS 2; yet, it showed a significant improvement in the POMS 2 Tension–Anxiety score and a significant reduction in the salivary CgA concentration without adverse effects. Hence, REP hydrolysate may be a helpful and harmless ingredient that can be used to relax anxiety symptoms and nervousness and prevent future related symptoms.

## Figures and Tables

**Table 1:** Background characteristics of participants.

Parameter	Total (*n* = 76)	Placebo (*n* = 38)	Active (*n* = 38)
Age (years)	50.1 ± 8.1	53.1 ± 6.3	48.7 ± 9.2
Height (cm)	164.74 ± 9.4	164.24 ± 8.69	165.08 ± 10.24
Weight (kg)	59.00 ± 10.92	59.74 ± 11.59	58.26 ± 10.32
BMI (kg/m^2^)	21.58 ± 2.48	21.90 ± 2.60	21.26 ± 2.34
Heart rate (bpm)	71.97 ± 10.50	71.37 ± 10.16	72.57 ± 10.93
POMS2 FI	63.1 ± 1.0	63.1 ± 1.1	63.1 ± 0.9
POMS2 VA	37.2 ± 0.5	37.2 ± 0.5	37.2 ± 0.5
Euthymia Scale	3.5 ± 0.2	3.5 ± 0.3	3.4 ± 0.3

POMS, Profile of Mood Status; FI, Fatigue–Inertia; VA, Vigor–Activity. Values for the POMS 2 and Euthymia Scale were measured at the third pre-examination point. Other values were measured at the first pre-examination point. The baseline score of the POMS2 and Euthymia Scale are shown as the mean ± SEM. The other baseline parameters are shown as the mean ± S.D. *p* value: inter-group comparison with independent *t*-test.

**Table 2:** Overall study schedule.

	First Pre-Examination	Second Pre-Examination	Third Pre-Examination	0 Weeks (Study 1)	4 Weeks	Followup
Health check	●			●		●
Saliva collection				●		●
POMS 2		●	●	●	●	
Euthymia Scale		●	●	●	●	
ISI				●	●	

POMS, Profile of Mood Status; ISI, Insomnia Severity Index. Each measurement was performed on the days with circles (●). ISI at 0 week was conducted before the test food intake as the baseline of Study 2. The test food was consumed once a day over the 4-week study period (highlighted).

**Table 3:** Results of the POMS 2 in Study 1.

Subscales		Placebo (*n* = 38)		Active (*n* = 38)		*p* Value for Change
		Score	Change	Score	Change	
AH	Before	57.4 ± 1.9	−2.0 ± 1.4	53.3 ± 1.7	−2.9 ± 1.1	0.109
	After	55.4 ± 2.2		50.4 ± 1.9		
CB	Before	58.1 ± 1.7	−2.6 ± 1.0	60.2 ± 1.8	−4.3 ± 1.1	0.250
	After	55.5 ± 1.8		55.9 ± 2.3		
DD	Before	58.0 ± 2.0	−3.3 ± 0.8	60.3 ± 2.1	−2.6 ± 1.1	0.576
	After	54.7 ± 2.0		57.7 ± 2.2		
FI	Before	60.8 ± 1.5	−2.6 ± 1.0	60.3 ± 1.4	−4.6 ± 1.0	0.175
	After	58.2 ± 1.8		55.7 ± 1.8		
TA	Before	57.1 ± 1.4	−3.1 ± 0.8	58.5 ± 1.6	−5.8 ± 1.0	0.037
	After	53.9 ± 1.7		52.7 ± 2.2		
VA	Before	37.6 ± 0.8	+1.1 ± 0.5	37.1 ± 0.8	+0.0 ± 0.6	0.177
	After	38.6 ± 1.0		37.1 ± 0.9		
F	Before	40.6 ± 1.2	−0.1 ± 0.8	39.5 ± 1.0	+0.2 ± 0.8	0.801
	After	40.5 ± 1.3		39.7 ± 1.1		
TMD	Before	61.8 ± 1.6	−3.2 ± 0.8	61.9 ± 1.7	−4.3 ± 0.9	0.340
	After	58.7 ± 1.8		57.6 ± 2.1		

Values were shown as the mean ± SEM. POMS, Profile of Mood Status; AH, Anger–Hostility; CB, Confusion–Bewilderment; DD, Depression–Dejection; FI, Fatigue–Inertia; TA, Tension–Anxiety; VA, Vigor–Activity; F, Friendliness; TMD, Total Mood Disturbance. *p* value: inter-group comparison with independent *t*-test.

**Table 4:** Results of the Euthymia Scale in Study 1.

	Placebo (*n* = 38)		Active (*n* = 38)		*p* Value for Change
	Score	Change	Score	Change	
Before	3.9 ± 0.4	+0.7 ± 0.2	3.9 ± 0.4	+0.7 ± 0.3	0.882
After	4.6 ± 0.5		4.7 ± 0.4		

Values are shown as the mean ± SEM. *p* value: inter-group comparison with independent *t*-test.

**Table 5:** Salivary CgA concentrations in Study 1.

	Placebo (*n* = 30)		Active (*n* = 36)		*p* Value for Change
	pmol/mg Protein	Change	pmol/mg Protein	Change	
Before	9.0 ± 1.1	−0.8 ± 1.0	12.1 ± 1.7	−4.3 ± 1.2	0.134 *
After	8.3 ± 1.2		7.9 ± 0.9		0.034

Values are shown as the mean ± SEM. CgA; Chromogranin A. *p* value: inter-group comparison of concentration change with independent *t*-test. The *p* value with * indicates inter-group comparison of concentration at pre-intake.

**Table 6:** Results of the POMS 2 in Study 2.

Subscales	Weeks	Placebo (*n* = 38)		Active (*n* = 37)		*p* Value for Change
		Score	Change	Score	Change	
AH	0	57.9 ± 1.9	−6.5 ± 1.5	55.2 ± 1.8	−5.6 ± 1.6	0.674
	4	51.4 ± 1.6		49.7 ± 1.8		
CB	0	59.3 ± 2.0	−7.4 ± 1.4	61.6 ± 1.9	−7.9 ± 1.6	0.802
	4	51.9 ± 2.1		53.7 ± 2.3		
DD	0	59.8 ± 1.9	−6.8 ± 1.2	60.4 ± 2.1	−4.6 ± 1.3	0.244
	4	53.1 ± 2.0		55.8 ± 2.2		
FI	0	62.6 ± 1.6	−11.2 ± 1.7	63.1 ± 1.3	−9.2 ± 1.8	0.421
	4	51.5 ± 1.7		53.9 ± 1.9		
TA	0	58.2 ± 1.7	−6.9 ± 1.4	59.9 ± 1.6	−7.6 ± 1.8	0.781
	4	51.3 ± 2.0		52.4 ± 2.1		
VA	0	37.3 ± 0.8	+6.1 ± 1.1	37.2 ± 0.7	+3.6 ± 1.1	0.107
	4	43.4 ± 1.3		43.4 ± 1.3		
F	0	38.6 ± 1.0	+5.4 ± 1.4	39.5 ± 1.2	+2.4 ± 1.4	0.134
	4	44.0 ± 1.5		41.9 ± 1.3		
TMD	0	63.2 ± 1.6	−9.8 ± 1.4	63.4 ± 1.6	−8.2 ± 1.5	0.442
	4	53.5 ± 1.9		55.2 ± 2.1		

Values are shown as the mean ± SEM. POMS, Profile of Mood Status; AH, Anger–Hostility; CB, Confusion–Bewilderment; DD, Depression–Dejection; FI, Fatigue–Inertia; TA, Tension–Anxiety; VA, Vigor–Activity; F, Friendliness; TMD, Total Mood Disturbance. *p* value: inter-group comparison with independent *t*-test.

**Table 7:** Results of the Euthymia Scale in Study 2.

	Weeks	Placebo (*n* = 38)		Active (*n* = 37)		*p* Value for Change
		Score	Change	Score	Change	
Euthymia Scale	0	3.5 ± 0.4	+2.8 ± 0.3	3.4 ± 0.4	+1.7 ± 0.4	0.034
	4	6.4 ± 0.5		5.1 ± 0.5		

Values were shown as the mean ± SEM. *p* value: inter-group comparison with independent *t*-test.

**Table 8:** Results of the ISI.

	Weeks	Placebo (*n* = 38)		Active (*n* = 37)		*p* Value for Change
		Score	Change	Score	Change	
ISI	0	10.8 ± 0.9	−3.2 ± 0.7	11.9 ± 0.9	−3.3 ± 0.5	0.927
	4	7.6 ± 0.9		8.7 ± 0.8		

Values were shown as the mean ± SEM. ISI, Insomnia Severity Index. *P* value: inter-group comparison with independent *t*-test.

## Data Availability

Not applicable.
